# Covid-19 vaccination reported side effects and hesitancy among the Syrian population: a cross-sectional study

**DOI:** 10.1080/07853890.2023.2241351

**Published:** 2023-08-06

**Authors:** Michel Najjar, Sara Albuaini, Mohammad Fadel, Fatema Mohsen, Data Collection Group

**Affiliations:** Faculty of Medicine, Syrian Private University, Damascus, Syria

**Keywords:** War, crisis, low-income, immunization, adverse effects

## Abstract

**Introduction:**

Studying post-vaccination side effects and identifying the reasons behind low vaccine uptake are pivotal for overcoming the pandemic.

**Methods:**

This cross-sectional study was distributed through social media platforms and face-to-face interviews. Data from vaccinated and unvaccinated participants were collected and analyzed using the chi-square test, multivariable logistic regression to detect factors associated with side effects and severe side effects.

**Results:**

Of the 3509 participants included, 1672(47.6%) were vaccinated. The most common reason for not taking the vaccine was concerns about the vaccine’s side effects 815(44.4). The majority of symptoms were mild 788(47.1%), followed by moderate 374(22.3%), and severe 144(8.6%). The most common symptoms were tiredness 1028(61.5%), pain at the injection site 933(55.8%), and low-grade fever 684(40.9%). Multivariable logistic regression analysis revealed that <40 years (vs. ≥40; OR: 2.113, p-value = 0.008), females (vs. males; OR: 2.245, p-value< .001), did not receive influenza shot last year (vs. did receive Influenza shot last year OR: 1.697, p-value = 0.041), AstraZeneca (vs. other vaccine brands; OR: 2.799, p-value< .001), co-morbidities (vs. no co-morbidities; OR: 1.993, p-value = 0.008), and diabetes mellitus (vs. no diabetes mellitus; OR: 2.788, p-value = 0.007) were associated with severe post-vaccine side effects. Serious side effects reported were blood clots 5(0.3%), thrombocytopenia 2(0.1%), anaphylaxis 1(0.1%), seizures 1(0.1%), and cardiac infarction 1(0.1%).

**Conclusion:**

Our study revealed that most side effects reported were mild in severity and self-limiting. Increasing the public’s awareness of the nature of the vaccine’s side effects would reduce the misinformation and improve the public’s trust in vaccines. Larger studies to evaluate rare and serious adverse events and long-term side effects are needed, so people can have sufficient information and understanding before making an informed consent which is essential for vaccination.

## KEY MESSAGES

- Age < 40 years, females, not receiving influenza shot, AstraZeneca vaccine, co-morbidities, and diabetes mellitus were factors significantly associated with severe post-vaccination side effects.
- Although most of the reported vaccine side effects were mild in severity and well-tolerated, larger prospective studies to understand the causes of rare serious adverse events and long-term side effects are needed to overcome vaccine hesitancy among people and enable them to have sufficient information and understanding before making an informed consent which is essential for vaccination.

## Introduction

On 11 March 2020, the novel Coronavirus disease of 2019 (Covid-19) was announced a pandemic by the World Health Organization (WHO), only four months after the first known case of Covid-19, reported in China [1]. The pandemic spread has been rapid and heterogeneous causing one of the greatest global humanitarian crises in recorded history.

The vigilance around personal protective measures including wearing face masks, maintaining interpersonal 2-meter distance, avoiding mass gatherings, washing hands, and quarantine have slowly eased off. Although such measures might have mitigated the spread and thus saved lives, sustaining these measures in the long term must be balanced against the various health, social, and economic aspects [2]. Therefore, vaccines remain the only solution for this never-ending crisis, triggering a massive global effort [3], as leading pharmaceutical companies and scientists were in a race against time to develop and test the first effective vaccine. Several vaccines have been approved for emergency or full use by WHO: Pfizer–BioNTech, Oxford–AstraZeneca, Sinopharm BIBP, Moderna, Janssen, CoronaVac, Covaxin, Novavax, and Convidecia. Other vaccines are under assessment by the WHO: Sputnik V, Sinopharm WIBP, Abdala, Zifivax, Corbevax, V, COVIran Barekat, Sanofi–GSK, and SCB-2019 [4]. Immunization with the currently approved vaccines remains the only way to protect oneself against COVID-19, especially when there is no clear global consensus on the treatment guidinclusionnes for Covid-19.

Today 2.7 billion people have yet to receive their first vaccine dose against Covid-19 [5]. Most of the unvaccinated live in lower-middle-income and low-income countries [5]. In Syria, only 9.3% of the population is fully vaccinated [6], despite the total number of vaccines delivered to Syria by the Covid-19 Vaccines Global Access (COVAX) being sufficient to cover 39% of the population [7]. Syria received its first batch of Covid-19 vaccines on 21April 2021, delivered by COVAX [8]. The WHO supported the operating costs of vaccine administration, and vaccines are now available in 962 fixed sites: 39 hospitals, and 923 primary healthcare centres [7]. The vaccines currently available in Syria are Pfizer–BioNTech, AstraZeneca, Sinopharm, Moderna, Janssen, Sputnick light, Sputnick v, and Sinovac.

Although the efficacy and safety of the approved vaccines have been shown to be promising by many prestigious organizations [9,10], information on the long-term side effects and safety in literature remains scant. Headache, fever, fatigue, and pain at the site of injection were the most abundantly reported side effects of the Covid-19 vaccine, with the side effect severity ranging between mild and moderate [11,12,13]. Serious vaccine complications, despite the rarity, include blood clots after the first dose of the AstraZeneca vaccine [11]. This dangerous complication has negatively impacted the acceptance of vaccines and disseminated fear of Covid-19 vaccines. Despite Covid-19 infecting over half a billion and killing millions [14], the public’s lost confidence in vaccines safety and efficacy remains the main reason behind low vaccination rates [15,16]. The culprit, social media is negatively affecting the users’ views and intentions to enrol in the uptake of the vaccine [17,18]. For these reasons, post-vaccination surveillance of the vaccines’ side effects and reasons behind the stigma of vaccines has become an area of interest.

Our study aims to assess Covid-19 vaccine uptake, self-reported vaccine side effects and the reasons for not taking the vaccine among Syrians more than a year after the introduction of vaccines in Syria. The objectives of this study are to identify factors associated with Covid-19 vaccine side effects and severe side effects.

## Methods

### Study design and instrument

A cross-sectional study was carried out between 13 April 28 and May 2022, using convenience sampling strategy. A literature review was conducted to find related research on the topic of the study using relevant keywords (Covid-19 vaccines, side effects, Syria, and worldwide). After an extensive review, the survey was created by the authors according to the information published by the Centres for Disease Control and Prevention (CDC) [19]. We piloted the questionnaire to assess the clarity, acceptability, and relevance of the survey on 30 volunteers. These volunteers were happy with the questionnaire and were excluded from the final sample to avoid bias. The survey was displayed using Google Forms and distributed by the data collection group through social media apps (Facebook, Telegram, WhatsApp, and Instagram). To lower non-response bias, graphics interchange format (GIF) and posts were adapted to appeal to each social group. The questions were short multiple choice questions that required no typing. Authors interacted with the participants online and viewers were allowed to comment on the link to increase the survey’s ­popularity. Face-to-face interviews were also conducted with people in hospitals and on the streets. The inclusion criteria for this study were: Syrian citizens (residing in Syria or a Syrian who took the vaccine outside Syria), aged ≥ 16 years, willing to participate in the study, willing to complete the survey, and willing to provide informed consent. All other respondents not fulfilling the eligibility criteria were excluded from the study. Inability of the participants to complete any section or give contradictory answers rendered the response as incomplete and was then removed from the statistical analysis during validation of the responses. The time needed to complete the survey was 5 min. A brief description of the study was given before the participants and/or their legal guardian(s) provided informed consent. All participants were informed that their responses will remain confidential and will only be used for scientific purposes. The survey included three parts, the first part contained questions on demographic features including age, gender, health status, history of known allergy, history of seasonal influenza shot, smoking history, and history of Covid-19 infection. Confirmation of Covid-19 infection by polymerase chain reaction (PCR) was not required in this question. Previous studies found that self-reporting of Covid-19 related symptoms are adequate predictors of Covid-19 infection [20,21]. The second part had two options depending on whether the participant was vaccinated or not. Participants that did not take the vaccine were only asked for the reasons behind this, and then the survey would automatically end. For those that did take the vaccine, information gathered included the vaccine brand, number of doses received (Sputnik light, and Johnson and Johnson are single dose vaccines), vaccination dates, self-reported side effects post-vaccination, symptom severity (self-rated with the options mild, moderate, and severe), onset of symptoms, duration of symptoms, use of painkillers, need for medical assistance, serious adverse events, and history and date of post-vaccination Covid-19 infection. We also asked the participants about the symptoms that lasted more than two weeks post-vaccination. A sample size calculator (website: https://www.surveysystem.com/sscalc.htm) was used to calculate the sample size of 2401 participants based on a confidence interval of 2%, and a confidence level of 95%, for a population of 18284423.

### Statistical analysis

The data was collected *via* Google Forms and were analyzed by entering the data in the statistical package for the social sciences (SPSS) software version 25. Descriptive statistics were used to express the socio-demographic features of the study sample. Pearson Chi-Square test was performed to evaluate the association between categorical variables. All *p*-values < 0.05 were considered statistically significant. A multivariable logistic regression model was carried out to detect factors associated with side effects (vs. no side effects), the selected factors included age (<40 vs ≥40), gender (females vs males), smoking history (smoker vs non-smoker), occupation (healthcare worker vs non healthcare worker), seasonal influenza shot (received it vs did not receive it), vaccine brand (Oxford-AstraZenecaa vs other vaccine brands, Sputnik light vs other vaccine brands, and Pfizer-BioNTech vs other vaccine brands), history of Covid-19 infection (positive history vs no history of Covid-19 infection before vaccination), and co-morbidities (yes vs no). These selected factors entered into the model concurrently. The second multivariable logistic regression model was developed to identify factors associated with severe adverse effects (vs no, mild, and moderate side effects). the selected factors included age (<40 vs ≥40), gender (females vs males), smoking history (smoker vs non-smoker), occupation (healthcare worker vs non healthcare worker), seasonal influenza shot (received it vs did not receive it), vaccine brand (Oxford-AstraZenecaa vs other vaccine brands, Sputnik light vs other vaccine brands, and Pfizer-BioNTech vs other vaccine brands), history of Covid-19 infection (positive history vs no history of Covid-19 infection before vaccination), co-morbidities (yes vs no), diabetes mellitus (vs no diabetes mellitus), and hypertension (vs no hypertension).

## Results

### Demographic characteristics by vaccination status and severity of side effects

Of 3523 total participants who completed the survey, 14 participants who refused to be included in the study were excluded (completion rate = 99.6%). Of 3509 participants enrolled in the study, 1672 (47.6%) received at least one dose of the Covid-19 vaccine while 1837 (52.3%) remain unvaccinated. Of 1672 participants with at least one dose of Covid-19, 316 (18.9%) were partially vaccinated and 1356 (81.1%) were fully vaccinated. Males represented 1662 (47.4%) and females represented 1847 (52.6%) of the sample. The age group 20 to 29 years represented a majority in the sample 1380 (39.3%). Participants in the age category 30 to 59 years were lower in the vaccinated group 689 (41.2%) compared with the unvaccinated group 819 (44.6%) (*p*-value < .001). Regarding residency, people who live in rural areas were lower in the vaccinated 341 (20.4%) compared with the unvaccinated group 552 (30.0%) (*p*-value < .001). In contrast, Syrians who live in cities or who took the vaccine outside Syria were higher in the vaccinated group 1160 (69.4%) and 171 (10.2%) compared with the unvaccinated group 1244 (67.7%) and 41 (2.2%) (*p*-value < .001) respectively. Further data about participants residing outside Syria are available in the Supplementary Table 1. There were higher proportions of healthcare workers in the vaccinated group 660 (39.5%) compared with the unvaccinated group 380 (20.7%) (*p*-value < .001). Participants with a history of known allergy, chronic co-morbidity, and smoking were lower in the vaccinated group 208 (12.4%), 369 (22.1%), and 727 (43.5%) compared with the unvaccinated group 288 (15.7%), 459 (25.0), and 902 (49.1%) (*p*-value = 0.006, 0.042, and < 0.001) respectively. Participants who received an influenza shot last year were higher in the vaccinated group 406 (24.3%) compared with the unvaccinated group 87 (4.7%) (*p*-value < .001) (Table 1).

**Table 1.** Socio-demographic features by vaccination status and side effect severity.

Variables	All ParticipantsN = 3509 (%)	Vaccinated participantsn = 1672 (%)	Unvaccinated participantsn = 1837 (%)	*p*-value	No adverse effects *n* = 366 (%)	Mild adverse effects *n* = 788 (%)	Moderate adverse effects *n* = 374 (%)	Severe adverse effects *n* = 144 (%)	*p*-value
**Sex**									
Male	1662 (47.4)	788 (47.1)	874 (47.6)	0.790	214 (58.5)	376 (47.7)	158 (42.2)	40 (27.8)	<.001
Female	1847 (52.6)	884 (52.9)	963 (52.4)		152 (41.5)	412 (52.3)	216 (57.8)	104 (72.2)	
**Age (years)**									
16 - 20	427 (12.2)	145 (8.7)	282 (15.4)	<.001	28 (7.7)	73 (9.3)	28 (7.5)	16 (11.1)	<.001
20 - 29	1380 (39.3)	725 (43.4)	655 (35.7)		134 (36.6)	341 (43.3)	190 (50.8)	60 (41.7)	
30 - 39	658 (18.8)	307 (18.4)	351 (19.1)		53 (14.5)	156 (19.8)	69 (18.4)	29 (20.1)	
40 - 49	503 (14.3)	226 (13.5)	277 (15.1)		47 (12.8)	99 (12.6)	46 (12.3)	34 (23.6)	
50 - 59	347 (9.9)	156 (9.3)	191 (10.4)		54 (14.8)	73 (9.3)	25 (6.7)	4 (2.8)	
≥ 60	194 (5.5)	113 (6.8)	81 (4.4)		50 (13.7)	46 (5.8)	16 (4.3)	1 (0.7)	
**Residency**									
Syria (city)	2404 (68.5)	1160 (69.4)	1244 (67.7)	<.001					
Syria (village)	893 (25.4)	341 (20.4)	552 (30.0)						
Outside Syria	212 (6.0)	171 (10.2)	41 (2.2)						
**Occupation**									
Healthcare Worker	1040 (29.6)	660 (39.5)	380 (20.7)	<.001	132 (36.1)	322 (40.9)	155 (41.4)	51 (35.4)	0.260
**Co-morbidities**									
co-morbidities	828 (23.6)	369 (22.1)	459 (25.0)	0.042	78 (21.3)	156 (19.8)	79 (21.1)	56 (38.9)	<.001
Allergy	496 (14.1)	208 (12.4)	288 (15.7)	0.006	31 (8.5)	68 (8.6)	49 (13.1)	60 (41.7)	<.001
Hypertension	316 (9.0)	155 (9.3)	161 (8.8)	0.601	49 (13.4)	67 (8.5)	27 (7.2)	12 (8.3)	0.19
Diabetes Mellitus	191 (5.4)	84 (5.0)	107 (5.8)	0.296	21 (5.7)	27 (3.4)	16 (4.3)	20 (13.9)	0.00
Cardiovascular Disease	139 (4.0)	60 (3.6)	79 (4.3)	0.280	10 (2.7)	23 (2.9)	7 (1.9)	7 (4.9)	0.325
Gastrointestinal Disease	118 (3.4)	47 (2.8)	71 (3.9)	0.084	10 (2.7)	20 (2.5)	13 (3.5)	13 (9.0)	0.001
Respiratory Disease	112 (3.2)	56 (3.3)	56 (3.0)	0.613	9 (2.5)	30 (3.8)	13 (3.5)	8 (5.6)	0.379
Neurological Disease	67 (1.9)	20 (1.2)	47 (2.6)	0.003	0 (0.0)	2 (0.3)	5 (1.3)	4 (2.8)	0.001
Hematological Disease	35 (1.0)	11 (0.7)	24 (1.3)	0.053	0 (0.0)	1 (0.1)	0 (0.0)	1 (0.7)	0.181
Liver Disease	7 (0.2)	2 (0.1)	5 (0.3)	0.312	5 (1.4)	13 (1.6)	8 (2.1)	4 (2.8)	0.678
Autoimmune Disease	49 (1.4)	30 (1.8)	19 (1.0)	0.055	6 (1.6)	10 (1.3)	4 (1.1)	0 (0.0)	0.487
Renal diseases	21 (0.6)	8 (0.5)	13 (0.7)	0.379	1 (0.3)	0 (0.0)	3 (0.8)	0 (0.0)	0.065
Malignancies	16 (0.5)	4 (0.2)	12 (0.7)	0.069	78 (21.3)	156 (19.8)	79 (21.1)	56 (38.9)	<.001
Smoker									
Cigarettes and Shisha	1629 (46.4)	727 (43.5)	902 (49.1)	0.001	136 (37.2)	352 (44.7)	172 (46.0)	67 (46.5)	0.048

The 1837 unvaccinated participants reported the following reasons for not taking the vaccine: concern about the vaccines side effects 815 (44.4%), unconvinced of the vaccine benefits 762 (41.5%), will not contract Covid-19 as previously contracted the virus 400 (21.8%), medical exemption 94 (5.1%), and vaccine unavailability 84 (4.6%).

Of the 1672 vaccinated participants, 788 (47.1%) had mild side effects, 374 (22.3%) had moderate side effects, 366 (21.9%) had no side effects, and 144 (8.6%) had severe side effects. Regarding gender, a higher proportion of females suffered from severe side effects 104 (72.2%) compared with males 214 (58.5%) (*p*-value < .001). Reported severe side effects was higher among participants in the age categories 16–20 years 16 (11.0%), 20–29 years 60 (8.3%), 30–39 years 29 (9.5%), and 40–49 years 34 (15.0%) compared with the age categories 50–59 years 4 (2.6%) and 60≤ years 1 (0.9%) (*p*-value < .001) (Table 1).

Participants with chronic co-morbidities reported higher numbers of mild side effects 156 (42.3%) compared with no side effects 78 (21.1%) (*p*-value < .001). Of the co-morbidities reported, diabetes mellitus was associated with higher numbers of mild side effects 27 (32.1%) compared with no side effects 21 (25.0%) (*p*-value < .001), while allergies, respiratory disease, and hematological disease were associated with higher numbers of severe side effects 60 (28.8%), 13 (23.2%), and 4 (36.4%) compared with no side effects 31 (14.9%), 10 (17.9%), and (0.0%) (*p*-value < .001), (*p*-value = 0.001), and (*p*-value = 0.001), respectively (Table 1).

The majority 591 (81.3%) of smokers reported post-vaccine side effects compared with no side effects 136 (18.7%) (*p*-value = 0.048). A history of Covid-19 infection was associated with higher numbers of vaccine side effects 583 (82.6%) compared with no side effects 123 (17.4%) (*p*-value = 0.001). Participants who received an influenza vaccine (flu jab) reported higher numbers of mild side effects 192 (47.3%) and no side effects 133 (32.8%) compared with moderate side effects 60 (14.8%) and severe side effects 21 (5.2%) (*p*-value < .001) (Table 1).

### Covid-19 vaccine side effects and complications by vaccine brand

Vaccine brands available in Syria at the time of the study were AstraZeneca-Oxford 552 (33.0%), Sputnik light 294 (17.5%), Pfizer-BioNTech 280 (16.7%), Sputix v 203 (12.1%), Sinophram 140 (8.4%), Sinovac 93 (5.6%), Johnson & Johnson 58 (3.5%), and Moderna 52 (3.1%). The majority of symptoms started within 12 to 24 h 614 (47.0%) while the minority started after 48 h 36 (2.8%) (*p*-value = 0.001) (Table 2).

**Table 2.** Covid-19 vaccine side effects by vaccine brand.

	All vaccinated participants*n* = 1672 (%)	AstraZeneca-Oxford *n* = 552 (%)	- Pfizer BioNTech*n* = 280 (%)	Sputnik light *n* = 294 (%)	Sputnik v*n* = 203 (%)	Sinopharm*n* = 140 (%)	Sinovac*n* = 93 (%)	Johnson & Johnson*n* = 58 (%)	Moderna*n* = 52 (%)	p-value
**Onset of symptoms**										
Within 12 hrs	555 (42.5)	215 (47.6)	79 (37.4)	106 (44.4)	62 (38.0)	28 (31.5)	27 (43.5)	21 (41.2)	17 (43.6)	0.001
12 to 24 hrs	614 (47.0)	194 (42.9)	114 (54.0)	97 (40.6)	90 (55.2)	48 (53.9)	25 (40.3)	25 (49.0)	21 (53.8)	
24 to 48 hrs	101 (7.7)	31 (6.9)	13 (6.2)	30 (12.6)	11 (6.7)	8 (9.0)	4 (6.5)	3 (5.9)	1 (2.6)	
After 48 hrs	36 (2.8)	12 (2.7)	5 (2.4)	6 (2.5)	0 (0.0)	5 (5.6)	6 (9.7)	2 (3.9)	0 (0.0)	
**Symptoms**										
Tiredness & Fatigue	1028 (61.5)	377 (68.3)	156 (55.7)	200 (68.0)	120 (59.1)	67 (47.9)	39 (41.9)	38 (65.5)	31 (59.6)	<.001
Headache	648 (38.8)	254 (46.0)	96 (34.3)	114 (38.8)	74 (36.5)	36 (25.7)	23 (24.7)	24 (41.4)	27 (51.9)	<.001
Low grade Fever (<39)	684 (40.9)	236 (42.8)	103 (36.8)	141 (48.0)	77 (37.9)	46 (32.9)	26 (28.0)	32 (55.2)	23 (44.2)	0.001
High grade Fever (>39)	175 (10.5)	90 (16.3)	14 (5.0)	16 (5.4)	24 (11.8)	10 (7.1)	8 (8.6)	6 (10.3)	7 (13.5)	<.001
Chills	334 (20.0)	157 (28.4)	38 (13.6)	49 (16.7)	47 (23.2)	16 (11.4)	5 (5.4)	16 (27.6)	6 (11.5)	<.001
Pain at the injection site	933 (55.8)	347 (62.9)	148 (52.9)	190 (64.6)	110 (54.2)	48 (34.3)	31 (33.3)	29 (50.0)	30 (57.7)	<.001
Swelling/redness/temperature at the injection site	305 (18.2)	108 (19.6)	60 (21.4)	45 (15.3)	46 (22.7)	17 (12.1)	5 (5.4)	9 (15.5)	15 (28.8)	<.001
Itchy or irritation in skin	61 (3.6)	24 (4.3)	14 (5.0)	9 (3.1)	7 (3.4)	3 (2.1)	3 (3.2)	0 (0.0)	1 (1.9)	0.526
Joints pain	513 (30.7)	215 (38.9)	65 (23.2)	83 (28.2)	53 (26.1)	31 (22.1)	25 (26.9)	25 (43.1)	16 (30.8)	<.001
Myalgia/ Muscle Pain	615 (36.8)	261 (47.3)	89 (31.8)	103 (35.0)	68 (33.5)	30 (21.4)	25 (26.9)	21 (36.2)	18 (34.6)	<.001
Abdominal Pain	109 (6.5)	50 (9.1)	15 (5.4)	10 (3.4)	14 (6.9)	8 (5.7)	5 (5.4)	2 (3.4)	5 (9.6)	0.066
Nausea/ Vomiting	124 (7.4)	54 (9.8)	19 (6.8)	14 (4.8)	14 (6.9)	4 (2.9)	9 (9.7)	6 (10.3)	4 (7.7)	0.062
Diarrhea	57 (3.4)	23 (4.2)	13 (4.6)	2 (0.7)	5 (2.5)	2 (1.4)	3 (3.2)	7 (12.1)	2 (3.8)	0.001
Skin rashes	15 (0.9)	7 (1.3)	2 (0.7)	0 (0.0)	4 (2.0)	0 (0.0)	1 (1.1)	0 (0.0)	1 (1.9)	0.275
Blurred vision	39 (2.3)	22 (4.0)	8 (2.9)	2 (0.7)	1 (0.5)	2 (1.4)	3 (3.2)	0 (0.0))	1 (1.9)	0.028
Ankle or feet swelling	20 (1.2)	13 (2.4)	3 (1.1)	1 (0.3)	1 (0.5)	0 (0.0)	1 (1.1)	0 (0.0)	1 (1.9)	0.114
Body bruises	15 (0.9)	8 (1.4)	2 (0.7)	1 (0.3)	1 (0.5)	0 (0.0)	1 (1.1)	1 (1.7)	1 (1.9)	0.568
Bleeding gums	8 (0.5)	2 (0.4)	3 (1.1)	1 (0.3)	0 (0.0)	0 (0.0)	1 (1.1)	0 (0.0)	1 (1.9)	0.404
Epistaxis	6 (0.4)	1 (0.2)	2 (0.7)	1 (0.3)	0 (0.0)	0 (0.0)	1 (1.1)	0 (0.0)	1 (1.9)	0.349
Sweating	319 (19.1)	136 (24.6)	39 (13.9)	46 (15.6)	37 (18.2)	18 (12.9)	12 (12.9)	19 (32.8)	12 (23.1)	<.001
Cough	178 (10.6)	67 (12.1)	28 (10.0)	20 (6.8)	17 (8.4)	14 (10.0)	7 (7.5)	11 (19.0)	14 (26.9)	<.001
Nasal congestion	197 (11.8)	97 (17.6)	24 (8.6)	21 (7.1)	22 (10.8)	12 (8.6)	7 (7.5)	8 (13.8)	6 (11.5)	<.001
Runny nose	133 (8.0)	59 (10.7)	20 (7.1)	10 (3.4)	16 (7.9)	11 (7.9)	6 (6.5)	8 (13.8)	3 (5.8)	0.014
Sore throat	168 (10.0)	76 (13.8)	26 (9.3)	19 (6.5)	19 (9.4)	9 (6.4)	8 (8.6)	7 (12.1)	4 (7.7)	0.028
Laziness	607 (36.3)	247 (44.7)	92 (32.9)	112 (38.1)	55 (27.1)	39 (27.9)	18 (19.4)	22 (37.9)	22 (42.3)	<.001
Sleepiness or insomnia	416 (24.9)	160 (29.0)	60 (21.4)	87 (29.6)	44 (21.7)	24 (17.1)	16 (17.2)	11 (19.0)	14 (26.9)	0.006
Irregular heart beats	114 (6.8)	64 (11.6)	8 (2.9)	18 (6.1)	8 (3.9)	2 (1.4)	5 (5.4)	5 (8.6)	4 (7.7)	<.001
Increase or decrease in blood pressure	77 (4.6)	44 (8.0)	7 (2.5)	8 (2.7)	8 (3.9)	1 (0.7)	4 (4.3)	0 (0.0)	5 (9.6)	<.001
Chest pain	70 (4.2)	38 (6.9)	5 (1.8)	9 (3.1)	3 (1.5)	4 (2.9)	5 (5.4)	5 (8.6)	1 (1.4)	0.002
Dyspnea	120 (7.2)	57 (10.3)	12 (4.3)	20 (6.8)	10 (4.9)	5 (3.6)	9 (9.7)	5 (8.6)	2 (3.8)	0.012
Anxiety	82 (4.9)	43 (7.8)	11 (3.9)	10 (3.4)	3 (1.5)	0 (0.0)	5 (5.4)	8 (13.8)	2 (3.8)	<.001
Loss of consciousness	10 (0.6)	6 (1.1)	0 (0.0)	1 (0.3)	0 (0.0)	0 (0.0)	1 (1.1)	1 (1.7)	1 (1.9)	0.224
**Severity of Symptoms**										
Asymptomatic	366 (21.9)	100 (18.1)	69 (24.6)	55 (18.7)	40 (19.7)	51 (36.4)	31 (33.3)	7 (12.1)	13 (25.0)	<.001
Mild	844 (52.9)	215 (38.9)	138 (49.3)	168 (57.1)	103 (50.7)	68 (48.6)	46 (49.5)	26 (44.8)	24 (46.2)	<.001
Moderate	374 (22.4)	154 (27.9)	58 (20.7)	58 (19.7)	49 (24.1)	16 (11.4)	8 (8.6)	21 (36.2)	10 (19.2)	<.001
Severe	144 (8.6)	83 (15.0)	15 (5.4)	13 (4.4)	11 (5.4)	5 (3.6)	8 (8.6)	4 (6.9)	5 (9.6)	<.001
**Duration of symptoms**										
Less than 12 hrs	262 (20.1)	81 (17.9)	48 (22.7)	48 (20.1)	35 (21.5)	18 (20.2)	21 (33.9)	6 (11.8)	5 (12.8)	0.011
12 to 24 hrs	508 (38.9)	151 (33.4)	79 (37.4)	107 (44.8)	67 (41.1)	38 (42.7)	22 (35.5)	27 (52.9)	17 (43.6)	
1 day to 2 days	380 (29.1)	147 (32.5)	61 (28.9)	65 (27.2)	50 (30.7)	24 (27.0)	10 (16.1)	11 (21.6)	12 (30.8)	
3 days to 1 week	105 (8.0)	47 (10.4)	17 (8.1)	13 (5.4)	8 (4.9)	6 (6.7)	3 (4.8)	6 (11.8)	5 (12.8)	
1 to 2 weeks	23 (1.8)	11 (2.4)	2 (0.9)	3 (1.3)	2 (1.2)	3 (3.4)	2 (3.2)	0 (0.0)	0 (0.0)	
More than 2 weeks	28 (2.1)	15 (3.3)	4 (1.9)	3 (1.3)	1 (0.6)	0 (0.0)	4 (6.5)	1 (2.0)	0 (0.0)	
**Management**										
use of painkillers	1001 (59.9)	377 (68.3)	139 (49.6)	182 (61.9)	122 (60.1)	69 (49.3)	42 (45.2)	39 (67.2)	31 (59.6)	<.001
Require Hospitalization	65 (3.9)	34 (6.2)	9 (3.2)	6 (3.1)	6 (3.0)	2 (1.4)	3 (3.2)	1 (1.7)	1 (1.9)	0.082
**Serious medical condition**										
Blood clot	5 (0.3)	2 (0.4)	1 (0.4)	1 (0.3)	0 (0.0)	0 (0.0)	1 (1.1)	0 (0.0)	0 (0.0)	0.850
Low platelets count	2 (0.1)	1 (0.2)	0 (0.0)	1 (0.3)	0 (0.0)	0 (0.0)	0 (0.0)	(0.0)	0 (0.0)	0.937
Anaphylaxis	1 (0.1)	1 (0.2)	0 (0.0)	0 (0.0)	0 (0.0)	0 (0.0)	0 (0.0)	0 (0.0)	0 (0.0)	0.958
Seizures	1 (0.1)	1 (0.2)	0 (0.0)	0 (0.0)	0 (0.0)	0 (0.0)	0 (0.0)	0 (0.0)	0 (0.0)	0.958
Cardiac infarction	1 (0.1)	1 (0.2)	0 (0.0)	0 (0.0)	0 (0.0)	0 (0.0)	0 (0.0)	0 (0.0)	0 (0.0)	0.958
**Covid-19 infection**										
Covid-19 infection after first dose	129 (7.7)	42 (7.6)	20 (7.1)	31 (10.5)	11 (5.4)	11 (7.9)	12 (12.9)	2 (3.4)	0 (0.0)	<.001
Covid-19 infection after second dose	117 (7.0)	38 (6.9)	27 (9.6)	0 (0.0)	26 (12.8)	21 (15.0)	5 (5.4)	0 (0.0)	0 (0.0)	
Covid-19 vaccine side effects by type of formulation										
	Viral-vector vaccines*n* = 1107 (%)	mRNA vaccines *n* = 332 (%)	Inactivated vaccine *n* = 233 (%)	p-value						
**Post vaccination symptoms**										
Symptomatic	905 (81.8)	250 (75.3)	151 (64.8)	<.001						
Mild symptoms	512 (46.3)	162 (48.8)	114 (48.9)							
Moderate symptoms	282 (25.5)	68 (20.5)	24 (10.3)							
Severe symptoms	111 (10.0)	20 (6.0)	13 (5.6)							
**Duration of symptoms**										
Less than 12 hrs	170 (18.8)	53 (21.2)	39 (25.8)	0.360						
12 to 24 hrs	352 (38.9)	96 (38.4)	60 (39.7)							
1 day to 2 days	273 (30.2)	73 (29.2)	34 (22.5)							
3 days to 1 week	74 (8.2)	22 (8.8)	9 (0.7)							
1 to 2 weeks	16 (1.8)	2 (0.8)	5 (3.3)							
More than 2 weeks	4 (2.2)	4 (1.6)	4 (2.6)							
**Management**										
use of painkillers	720 (65.0)	170 (51.2)	111 (47.6)	<.001						
Require Hospitalization	50 (4.5)	10 (3.0)	5 (2.1)	0.154						
**Covid-19 infection**										
Covid-19 infection after first dose	86 (7.8)	20 (6.0)	23 (9.9)	0.013						
Covid-19 infection after second dose	64 (5.8)	27 (8.1)	26 (11.2)							

The most common reported side effects were tiredness and fatigue 1028 (61.5%), pain at the injection site 933 (55.8%), low-grade fever 684 (40.9%), headache 648 (38.8%), and muscle pain 615 (36. 8%). Tiredness and fatigue were higher among AstraZeneca-Oxford 377 (68.3%) and Sputnick light 200 (68.0%) compared with Sinophram 67 (47.9%) and Sinivac 39(41.9%) (*p*-value < .001). Headache was more common among Moderna 27 (51.9%) compared with Sinovac 23 (24.7%) (*p*-value < .001). Low grade fever (<39) was more common among Johnson & Johnson 32 (55.2%) compared with Sinovac 26 (28.0%) (*p*-value = 0.001). High grade fever was more common among AstraZeneca 90 (16.3%) compared with Pfizer-BioNTech 14 (5.0%) (*p*-value < .001). Chills were higher among AstraZeneca 157 (28.4%) compared with Sinovac 5 (5.4%) (*p*-value < .001). Pain at the injection site was more common among Sputnik light 190 (64.6%) compared with Sinovac 31 (33.3%) (*p*-value < .001). Swelling, redness, and/or temperature at the injection site were more common among Moderna 15 (28.8%) compared with Sinovac 5 (5.4%) (*p*-value < .001). Joint pain was more common among Johnson & Johnson 25 (43.1%) compared with Pfizer-BioNTech 65 (23.2%) (*p*-value < .001). Myalgia was more common among AstraZeneca 261 (47.3%) compared with Sinopharm 30 (21.4%) (*p*-value < .001). Diarrhoea was more common among Johnson & Johnson 7 (12.1%) compared with Sputnick light 2 (0.7%) (*p*-value = 0.001). Blurred vision was higher among AstraZeneca 22 (4.0%) compared with Johnson & Johnson 0 (0.0%) (*p*-value = 0.028). Sweating was more common among Johnson & Johnson 19 (32.8%) compared with Sinopharm 18 (12.9%) and Sinovac 12 (12.9%) (*p*-value < .001). Cough was more common among Moderna 14 (26.9%) compared with Sputnick light 20 (6.8%) (*p*-value < .001). Nasal congestion was more common among AstraZeneca 97 (17.6%) compared with Sputnick light 21 (7.1%) (*p*-value < .001). Runny nose was more common among Johnson & Johnson 8 (13.8%) compared with Sputnick light 10 (3.4%). Sore throat was more common among AstraZeneca 76 (13.8%) compared with Sinopharm 9 (6.4%) (*p*-value = 0.028). Laziness was more common among AstraZeneca 247 (44.7%) compared with Sinovac 18 (19.4%) (*p*-value < .001). Insomnia was more common among Sputnick light 87 (29.6%) compared with Sinopharm 24 (17.1%) (*p*-value = 0.006). Dysrhythmia was more common among AstraZeneca 64 (11.6%) compared with Sinopharm 2 (1.4%). Hypotension or hypertension was more common among Moderna 5 (9.6%) compared with Johnson & Johnson 0 (0.0%) (*p*-value < .001). Chest pain was more common among Johnson & Johnson 5 (8.6%) compared with Moderna 1 (1.4%) (*p*-value = 0.002). Dyspnoea was more common among AstraZeneca 57 (10.3%) compared with Sinopharm 5 (3.6%) (*p*-value = 0.012). Anxiety was more common among Johnson & Johnson 8 (13.8%) compared with Sinopharm 0 (0.0%) (*p*-value < .001) (Table 2).

**Figure 1.**
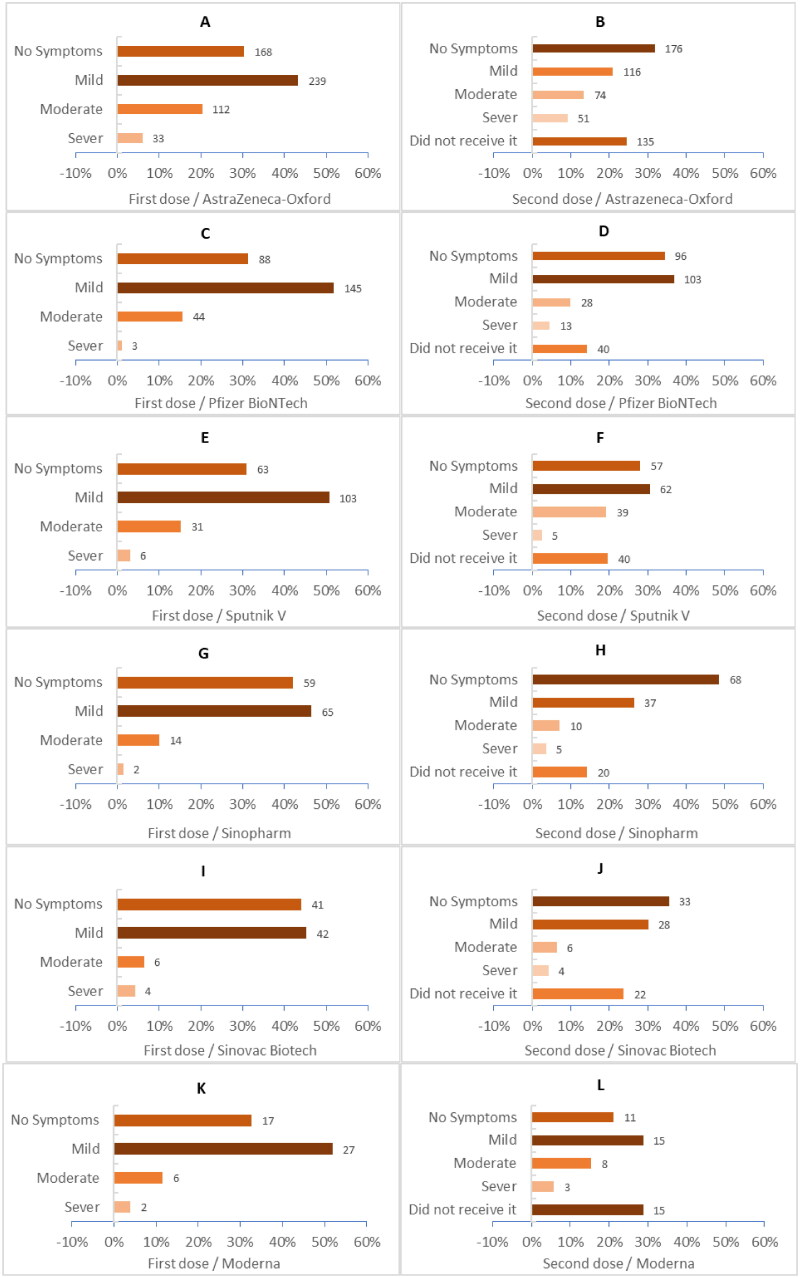
(A to L) Shows the side effect severity after receiving each dose.

Regarding Covid-19 vaccine symptom severity, the Sinopharm vaccine was associated with a higher percentage of no side effects 51 (36.4%) compared with Johnson & Johnson 7 (12.1%) (*p*-value < .001), while most of the mild side effects were associated with Sputnik light 168 (57.1%) compared with AstraZeneca 215 (38.9%) (*p*-value < .001). Moderate side effects were most reported among participants who received Johnson & Johnson 21 (36.2%) compared with Sinovac 8 (8.6%) (*p*-value < .001). Severe side effects were most associated with AstraZeneca 83 (15.0%) compared with Sinopharm 5 (3.6%) (*p*-value < .001) (Table 2). Post-vaccination side effect severity varied across the first and second doses (Figure 1). Shockingly, severe side effects were higher after the second dose of most vaccines, including AstraZeneca, Pfizer-BioNTech, Sinopharm, and Moderna (Figure 1 B, D, H, and L).

The duration of post-vaccination symptoms were reported as follows, >12 h 262 (20.1%), 12-24 h 508 (38.9%), 1-2 days 380 (29.1%), 3 days to 1 week 105 (8.0%), 1-2 weeks 23 (1.8%), and >2 weeks 28 (2.1%) (*p*-value = 0.011) (Table 2). Participants, whose symptoms lasted over 2 weeks, self-reported the following symptoms, pain at the injection site 20 (1.2%), muscle or joint pain 13 (0.8%), fatigue 11 (0.7%), dysrhythmia 5 (0.3%), menstrual abnormalities 4 (0.5%), headache 3 (0.2%), and nasal congestion 2 (0.1%). Other symptoms include low white blood cell count and low platelet count, swollen legs, leg purpura, and hypertension, which were each reported among 1 (0.1%) participant.

The proportion of participants who took painkillers for their symptoms were higher among AstraZeneca 377 (68.3%), Johnson & Johnson 39 (67.2%), Sputnik Light 182 (61.9%), Sputnik v 122 (60.1%), and Moderna 31 (59.6%) compared with Pfizer-BioNTech 139 (49.6%), Sinopharm 69 (49.3%), and Sinovac 42 (45.2%). The highest number of required hospitalization post-vaccination was among AstraZeneca 34 (6.2%) (Table 2).

Serious medical complications, such as blood clots and low platelet counts were reported by 5 (0.3%) and 2 (0.1%) respectively. Anaphylaxis shock 1 (0.1%), seizures 1 (0.1%), and cardiac infarction 1 (0.1%) were only reported among those who took the AstraZeneca vaccine (Table 2).

Regarding vaccine efficacy, a minority reported having Covid-19 infection after taking the first dose 129 (7.7%) and after taking two doses 117 (7.0%) (*p*-value < .001). The highest Covid-19 reinfection rate after the first dose was among Sinovac 12 (12.9%), whereas after the second dose was among Sinopharm 21 (15.0%) (*p*-value < .001) (Table 2).

Comparing Covid-19 vaccines by type of formulation (mRNA, inactivated, and viral-vector), showed that viral-vector vaccines were associated with a higher percentage of side effects 905 (81.8%) compared with m-RNA vaccines 250 (75.3%) and inactivated vaccines which were associated with lowest post vaccination side effects 151(64.8%) (*p*-value < .001). Viral- vector vaccines were also associated with higher percentage of severe side effects and use of painkillers 111 (10.0%) and 720 (65.0%) respectively compared to other types of formation (*p*-value = < 0.001). The highest reported Covid-19 reinfection rate after the first dose and the second dose were among inactivated vaccines 23 (9.9%) and 26 (11.2%) retrospectively (*p*- value = 0.013) (Table 2).

### Multivariate logistic regression analysis

A multivariate logistic regression analysis was performed to identify the variables including age, sex, smoking, occupation, influenza vaccine, vaccine brand, history of Covid-19 infection, and co-morbidities and their association with the development of side effects versus no side effects post-vaccination. The logistic regression model was statistically significant for the following factors, age <40 (vs. ≥40; OR: 1.866, *p*-value < .001), females (vs. males; OR: 1.696, *p*-value < .001), current smoker (vs. non-smoker; OR: 1.428, *p*-value = .006), did not receive Influenza shot last year (vs. did receive Influenza shot last year OR: 1.929, *p*-value < .001), AstraZeneca vaccine (vs. other vaccine brands OR: 1.426, *p*-value = .021), history of Covid-19 infection pre-vaccination (vs. no history of Covid-19 infection pre-vaccination OR: 1.317, *p*-value = .034), and co-morbidities (vs. no co-morbidities, OR: 1.438, *p*-value .029) were significantly associated with post-vaccination side effects (Table 3).

**Table 3.** Multivariate logistic regression analysis on variables associated with side effects and severe side effects after Covid-19 vaccination.

Factors	OR	95% C.I. for 0R		*p*-value
		Lower	Upper	
Side Effects				
Age <40 (vs. ≥40)	1.866	1.398	2.492	<0.001
Females (vs. males)	1.696	1.324	2.172	<0.001
Current smoker (vs. nonsmoker)	1.428	1.108	1.841	0.006
Healthcare worker (vs. non healthcare worker)	1.181	0.908	1.536	0.214
Did not receive Influenza shot last year (vs. did receive Influenza shot last year)	1.929	1.483	2.511	<0.001
Oxford-AstraZeneca (vs. other vaccine brands)	1.426	1.055	1.928	0.021
Sputnik light (vs. other vaccine brands)	1.283	0.890	1.850	0.181
Pfizer-BioNTech (vs. other vaccine brands)	1.050	0.742	1.485	0.783
History of Covid-19 infection pre-vaccination (vs. no history of Covid-19 infection pre-vaccination)	1.317	1.021	1.699	0.034
Co-morbidities (vs. no co-morbidities)	1.438	1.038	1.990	0.029
Severe Side Effects				
Age <40 (vs. ≥40)	2.113	1.214	3.678	0.008
Females (vs. males)	2.245	1.487	3.391	<0.001
Current smoker (vs. non smoker)	1.230	0.839	1.803	0.289
Healthcare worker (vs. non healthcare worker)	0.793	0.524	1.201	0.273
Did not receive Influenza shot last year (vs. did receive Influenza shot last year)	1.697	1.022	2.819	0.041
Oxford-AstraZeneca (vs. other vaccine brands)	2.799	1.767	4.435	<0.001
Sputnik light (vs. other vaccine brands)	0.629	0.315	1.255	0.188
Pfizer-BioNTech (vs. other vaccine brands)	1.008	0.517	1.967	0.981
History of Covid-19 infection pre-vaccination (vs. no history of Covid-19 infection pre-vaccination)	1.144	0.781	1.676	0.489
Co-morbidities (vs. no co-morbidities)	1.993	1.195	3.325	0.008
Hypertension (vs. no hypertension)	2.023	0.928	4.408	0.076
Diabetes Mellitus (vs. no diabetes mellitus)	2.788	1.316	5.909	0.007

A multivariate logistic regression analysis was performed to identify the variables including age, sex, smoking, occupation, influenza vaccine, vaccine brand, history of Covid-19 infection, co-morbidities, hypertension, and diabetes mellitus and their association with the development of severe side effects versus no, mild, and moderate side effects post-vaccination. The logistic regression model was statistically significant for the following factors, age <40 (vs. ≥40; OR: 2.113, *p*-value = 0.008), females (vs. males; OR: 2.245, *p*-value < .001), did not receive influenza shot last year (vs. did receive Influenza shot last year OR: 1.697, *p*-value = 0.041), AstraZeneca (vs. other vaccine brands; OR: 2.799, *p*-value < .001), co-morbidities (vs. no co-morbidities; OR: 1.993, *p*-value = 0.008), and diabetes mellitus (vs. no diabetes mellitus; OR: 2.788, *p*-value = 0.007) were significantly associated with severe post-vaccination side effects (Table 3).

## Discussion

Multiple vaccines have been developed during the past two years; these vaccines must be available, safe, and effective [22], with the aim to decrease the death and infection rates. Vaccine hesitancy represents a big obstacle despite all the efforts to counter the stigma [23]. Regarding vaccine status in Syria, vaccine hesitancy is reducing the vaccination prevalence among the population. In our study 47.6% of participants were vaccinated, much higher than the actual vaccination rate reported in the country (9.3%) [6]. The reason for this difference may be due to unreported bias, unvaccinated individuals refuse to express their opinion in a questionnaire distributed by healthcare providers due to medical mistrust of the medical system and Conspiracy theories [24]. A staggering 44.4% of unvaccinated participants reported that they were concerned about the vaccine’s side effects. Several studies in the United Kingdom and the United States of America showed that the main cause of vaccine hesitancy was concern about the side effects of the vaccines [25,26]. Despite, implementing various strategies to scale up vaccination campaigns and conduct vaccination at government institutions, universities, and schools, 4.6% of participants mentioned that they would like to take the vaccine, but the vaccine was not available. Syrians can receive the vaccine whether or not they are pre-registered through an online platform [7]. Our study showed that vaccine hesitancy was higher among rural areas, and this was consistent with previous Syrian studies [27,28]. A possible cause of this is the misunderstanding and myths regarding Covid-19 vaccines and conspiracy beliefs, which is more common in the Syrian countryside. Furthermore, we found that participants with a history of chronic co-morbidities or with known allergies were unwilling to get vaccinated. The fear of the vaccine side effects among those groups can be attributed to misinformation about Covid-19 vaccines in low-income countries [29]. Participants aged 30 to 59 were linked to the unvaccinated group. A study from Jordan indicated that the older age groups (>35 years old) were less likely to take the Covid-19 vaccines compared to younger age groups [30]. On the other hand, our data revealed higher vaccine acceptance among healthcare workers (HCW). In Syria, HCW were the first to receive priority access to vaccines [28]. HCW are also aware of the importance of vaccination to help protect them during occupational exposure and to prevent the spread of the disease among patients and the community [31]. Also, participants who received the influenza shot last year were linked to the vaccinated group and this was coherent with another study conducted in Jordan [30].

Regarding vaccinated participants, 78.1% were symptomatic after receiving a Covid-19 vaccine. The most common symptoms according to our results were tiredness and fatigue, pain at the injection site, low-grade fever, headache, and muscle pain. This was consistent with previous studies conducted in Saudi Arabia, Iraq, and the Czech Republic [32–34]. The symptoms were most frequently reported within 12 to 24 h after vaccination and lasted mainly one day. This result was in line with a study conducted in the Czech Republic and the United Kingdom [34,35]. The overwhelming majority of symptoms were mild and moderate in severity 64.6% and 28.6%, respectively. And this was consistent with what was announced by CDC and WHO [11,12]. Whereas, 8.6% of participants reported severe symptoms post-vaccination. A study from Jordan reported similar results [36]. However, an observational study in the United Arab Emirates and a cross-sectional study of healthcare workers in the United States of America showed various proportions of severe symptoms [13,37]. The majority of vaccinated participants used painkillers to alleviate post-vaccination discomfort; the highest proportion of those was among AstraZeneca-Oxford (ChAdOx1) vaccine recipients. Similar findings were observed in a study conducted in Togo [38]. In comparison between the first dose and second dose, we observed that the side effects tend to be more severe after receiving the second dose, specifically AstraZeneca-Oxford (ChAdOx1) and Pfizer BioNTech (BNT162b2). This was similar to previous studies which demonstrated that systematic and local side-effects were more common after receiving the second dose of Covid-19 vaccines [35,39], and coherent with what was announced by CDC [14]. Serious side effects after Covid-19 vaccination are rare but can occur [11], in our study 3.9% of vaccinated participants reported they required medical consultation or hospital visit. This was similar to a study conducted in the United Arab Emirates [13]. Serious side effects included blood clots, thrombocytopenia, anaphylaxis shock, seizures, and cardiac infarction. Despite the rarity of these serious side effects [40], and the lack of consensus on their association with vaccines [41], all of them have been previously mentioned in the medical literature [42–46].

In this study, people with a history of previous Covid-19 infection had greater odds of post-vaccine side effects. This finding was similar to previous studies conducted in the United Kingdom and Italy [44,47]. Participant characteristics of young age (<40 years), female sex, current smokers, history of chronic co-morbidities, AstraZeneca-Oxford vaccine, and did not receive influenza shot last year had greater odds of post-vaccination side effects. These results were consistent with data from a large cohort study in the United States of America [39]. Furthermore, this study revealed that participants with younger age (<40 years), female sex, history of chronic co-morbidities, AstraZeneca-Oxford vaccine, did not receive influenza shot last year also, and diabetes mellitus had greater odds of severe post-vaccination side effects. This finding was in line with previous studies conducted in Togo and Mexico [38,48]. Also, in this study participants with diabetes mellitus were more vulnerable to severe post-vaccination side effects. Another study found that patients at greatest risk of developing side effects post-vaccination include those with a history of type-2 diabetes [49].

Pain at the injection site was the most frequent symptom, and the reason behind this might be due to delayed-onset injection site reactions [40,50], or what is called ‘Covid arm’, which is a delayed but harmless allergic reaction [51,52]. Irregular heartbeats were reported by participants, although it is a rare prolonged side effect, a research study published in Nature Medicine looks at the possible link between different cardiac arrhythmias and Covid-19 vaccination [53]. Menstrual abnormalities were self-reported as a prolonged symptom, and many studies have shown a possible link between the Covid-19 vaccine and menstrual abnormalities [54,55].

Syrian vaccination rates are extremely low by global standards [6], but the explosion of Covid-19 infections has not been a problem which may be because the true scope of the Covid-19 outbreak in Syria is unknown due to limited testing capacity, underreporting, and lack of access to healthcare. However, a previous study showed that the increases in Covid-19 are unrelated to levels of vaccination across 68 countries and 2947 counties in the United States [56]. the highest infection rate after the first dose of Covid-19 vaccines was observed among the Sinovac (Coronavac) vaccine, which indicated that this vaccine provided poor immune protection against Covid-19. A previous study in Malaysia found that Coronavac effectiveness against Covid-19 infection waned after 3–5 months of full vaccination [57]. Sinopharm (BIBP) Vaccines had the highest infection rate after the second dose compared to all the other vaccine brands. A study from the Kingdom of Bahrain showed that compared to individuals vaccinated with AstraZeneca, Pfizer-BioNTech, or Sputnik V, those vaccinated with the Sinopharm vaccine had a higher risk of post-vaccination infection [58].

The cross -vaccine comparison of our study showed that viral vector-based vaccines were associated with the more frequent side effects. Our results are consistent with the findings of a German cross-sectional study [59]. A study from Algeria revealed that side effects are more prevalent among viral vector vaccines than inactivated virus vaccines [60], and this was adherent with our findings. In contrast, we found that inactivated virus vaccines were associated with lower adverse effects following vaccination. A systematic review study on the safety profile of covid-19 vaccines was showed that the low rates of local and systemic reactions were significantly lower among inactivated vaccines [61]. Inactivated virus vaccine showed higher reinfection rates after vaccination and this finding were consistent with other previous studies [62,63].

### Study limitations

The data deduced may not be generalized to the wider Syrian population. Credible published national data on the socio-demographic distribution of the population are unavailable to assess the representativeness of the study’s sample. The authors used a convenience sampling strategy involving various social media platforms and convenient location interviews. Syrians of an older age group represented a minority due to limited internet access. The elderly, the most vulnerable population, require vaccine protection; therefore, a study must be conducted to assess the vaccine uptake among this age group. As such, reaching out to these vulnerable populations must be prioritized. Additionally, as this study is a self-reported survey, the responses may be subject to recall bias.

## Conclusion

Most of the reported vaccine side effects were mild in severity and well-tolerated. However, age < 40 years, females, not receiving influenza shot, AstraZeneca vaccine, co-morbidities, and diabetes mellitus were factors significantly associated with severe post-vaccination side effects. Viral vector-based vaccines were associated with the more frequent side effects. Inactivated virus vaccines were associated with lower adverse effects and higher reinfection rates following vaccination. Larger prospective studies to understand the causes of rare serious adverse events are needed to overcome vaccine hesitancy among people. The long-term adverse effects of vaccines will become more important in the future. However, these adverse effects may not be known unless the medical environment is favourable and access to medical care is easy.

## Supplementary Material

Supplemental Material

## Data Availability

The data that support the findings of this study are openly available in [repository name ‘zenodo’] at https://doi.org/10.5281/zenodo.7425817 .
