# Supplementary material for: Covid-19 vaccination reported side effects and hesitancy among the Syrian population: a cross-sectional study
Source: Ann Med. 2023 Aug 6;55(2):2241351. doi: 10.1080/07853890.2023.2241351 (PMC10405764; doi:10.1080/07853890.2023.2241351)
Supplement: Supplemental Material [file IANN_A_2241351_SM7226.docx]

Supplementary

Table 1. Countries where Syrians took the vaccine

| Country name | Numbers of participants (%) |
| --- | --- |
| Germany | 54 (1.5) |
| UAE | 30 (0.9) |
| Lebanon | 23 (0.7) |
| KSA | 16 (0.5) |
| Kuwait | 7 (0.2) |
| Iraq | 6 (0.2) |
| Canada | 21 (0.6) |
| Turkey | 12 (0.3) |
| Yamen | 1 (0.0) |
| Belgium | 2 (0.1) |
| France | 4 (0.1) |
| Poland | 1 (0.0) |
| Russia | 3 (0.1) |
| Romania | 1 (0.0) |
| Egypt | 4 (0.1) |
| Jorden | 6 (0.2) |
| Venezuela | 3 (0.1) |
| USA | 4 (0.1) |
| Netherlands | 2 (0.1) |
| Bangladesh | 1 (0.0) |
| Oman | 2 (0.1) |
| Brazil | 1 (0.0) |
| Qatar | 3 (0.1) |
| Australia | 3 (0.1) |
| Sweden | 1 (0.0) |
| Malesia | 1 (0.0) |
